# Structure and driving factors of the soil microbial community associated with *Alhagi sparsifolia* in an arid desert

**DOI:** 10.1371/journal.pone.0254065

**Published:** 2021-07-09

**Authors:** Wenjing Li, Lamei Jiang, Yang Zhang, Dexiong Teng, Hengfang Wang, Jinlong Wang, Guanghui Lv

**Affiliations:** College of Resources and Environment Science, Xinjiang University, Urumqi, China; University of Salento, ITALY

## Abstract

Environmental properties are important factors in structuring soil microbial communities. The primary driving factors vary in different ecosystems. In the present work, we analyzed the microbial communities of rhizosphere and bulk soils associated with the halophyte *Alhagi sparsifolia* across three salt/water gradients in the desert area around Ebinur Lake Basin, China, using high-throughput sequencing technology. We found that there were significant differences in soil water content (SWC), soil salinity (SAL), total nitrogen (TN), and total phosphorus (TP) contents between the three water/salt gradients. In the L (low water and salt) plot, Actinobacteria was the most abundant bacterial phylum while Ascomycota was the dominant fungal phylum. The relative abundance of Actinobacteria was negatively correlated with soil pH, soil organic carbon (SOC), TP, and available phosphorus (AP). The abundance of Bacteroidetes was significantly positively correlated with soil SOC, SWC, SAL, pH, TN, and TP (*P* < 0.05). The abundance of fungal phylum Chytridiomycota was significantly positively correlated with pH (*P* < 0.01), SWC, AP, and sulfate ion (*P* < 0.05). SOC and nitrate nitrogen were the main factors impacting the bacterial community, while ammonium nitrogen (NH_4_^+^) and TP were the main driving forces for the fungal community. Soil nutrients were the main contributors to the dissimilarities in the bacterial and fungal communities, explaining 48.06% and 44.45% of the variation. SWC, SAL, and pH explained only a small percentage of the microbial community dissimilarity. In conclusion, soil microbial community structure was affected by SWC, SAL, pH, and soil nutrients, with soil nutrients as the main driving factors. Nitrogen has a differential effect on the different microbial communities: bacterial communities of *Alhagi sparsifolia* were mainly affected by nitrate nitrogen, while fungal communities were mainly driven by ammonium nitrogen.

## Introduction

Soil is the skin of the earth, supporting the main processes of life, maintaining ecosystem balance, and is also the home of a vast amount of microorganisms [1]. Soil microbial communities are the basis of soil ecological function, the main decomposers in terrestrial ecosystems, as well as the major participant and regulator of soil nutrient cycling and energy flows, which can be sensitive to and effective in predicting minor changes in soil ecosystems [2, 3]. Determining the composition of microbial communities is important in predicting the response of ecosystems to environmental changes [4, 5]. Therefore, it is imperative that we understand how exactly the soil environment shapes microbial community composition so that we can predict the ecosystem response to environmental change.

Soil factors play a key role in microbial community composition [6, 7]. In some research, scholars suggest that the soil microorganism communities of different ecosystem are shaped by different mechanisms. For example, in forest ecosystems, soil temperature and carbon-to-nitrogen ratio are the main driving forces of bacterial and fungal community structure [8]; in grassland ecosystems, the bacterial community is mainly determined by mutual plant-soil-microorganism interactions rather than directly driven by the changes in soil water content [9]; in wetland ecosystems, pH and conductivity have been considered to be the most important factors affecting bacterial community structure [10]. Arid land ecosystems are generally considered as fragile under global climate change, which makes them different from other ecosystems [11]. Soil water content is the main limiting factor for soil microorganisms in arid and semi-arid ecosystems, and the biomass and diversity of soil microorganisms will increase with the increase of soil water content [12–14]. Soil salinity has a significant impact on the diversity and structure of microbial communities in saline and alkaline environments [15, 16]. For bacterial communities, although the correlation between soil pH and bacterial community diversity is not significant, soil pH can change the spatial distribution of bacterial communities in arid and semi-arid areas by changing the individual abundance of bacterial communities [17]. It was found that the biomass of bacterial and fungal soil microbial communities in desert ecosystems is very low, with a significant positive correlation with soil organic carbon content [18–20]. Soil nutrients also affected the distribution pattern of individual group abundances in the bacterial community [17, 21]. Soil water, salt, pH, and soil nutrients play a key role in shaping the soil microbial community in desert ecosystems. However, the soil in the arid desert ecosystem is poor, and soil nutrients are an important limiting factor for microbial activities [22, 23].

Xinjiang is located in an inland arid area far from the sea. In addition, the terrain is closed, making it difficult for ocean water vapor to reach the area. This region is controlled by continental air masses throughout the year. The climate is dry and rainless. Plants in this region are mostly drought and salt tolerant. The desert area of the Ebinur Lake Basin is located in the northwest corner of Xinjiang. This area is the lowest depression and the center of water and salt accumulation along the southwest margin of Junggar basin [24]. Its unique topography has formed special water-salt transport characteristics in that salt comes in with water but also goes out with water, and water de-salts, which leads to serious salt accumulation and soil salinity in the area [25]. In saline-alkali soils, plants and microorganisms are subject to the dual restrictions of water and salinity. Due to long-term evolutionary adaptation, plants and microbiomes have gradually evolved salt and drought resistance. The structure of the soil microbial community in a particular habitat is affected by the factors such as the local climate and soil properties. The dominant factors and the role of each factor vary by habitat [26]. Previous studies focused on the effect of either the water or the salt gradient for soil microbial communities [27, 28], but did not reveal the change patterns of microbial communities along the dual gradients of water and salt in natural desert ecosystems. Understanding the effects of multiple soil factors on the structure and diversity of microbial communities in desert areas will help us understand the response and feedback capabilities of microorganisms to co-occurring environmental stressors in arid ecosystems.

*Alhagi sparsifolia* belongs to genus *Alhagi*, family *Leguminosae*, is mainly distributed in the desert areas of Eurasia, North America, North Africa, and Asia. It has highly developed deep roots, drought resistance, salt tolerance, soil stabilization, and potential for associated N_2_-fixing bacteria. In addition, this plant is important in the development of livestock husbandry due to its high protein content [29]. Studies on *Alhagi sparsifolia* mainly focus on the effects of addition of water and nitrogen on soil microbial community [30, 31] and analysis of root endosphere microbiomes [32]. However, the effect of changes in the microbial community structure on salt/water gradients have not been studied. The study goals were to: (1) analyze the diversity of bacterial and fungal microbial structure in the desert area of Ebinur lake and the changes along natural ecological gradients, in order to understand the effects of the potential responses of community structure and diversity of *Alhagi sparsifolia* to water/salt gradients; (2) determine the role environmental factors, such as SWC, SAL, pH, and soil nutrients, have on shaping microbial community structure; and (3) evaluate the relative contributions of nitrate nitrogen and ammonium nitrogen on different microbial groups.

## Materials and methods

### Study area

The Ebinur National Nature Reserve (44°30’–45°09’ N, 82°36’–83°50’ E) is located at the western margin of the Gurbantunggut Desert in Xinjiang, China, with a total area of 2670.85 km^2^. The climate of this region is a typical temperate continental arid climate, with an annual average precipitation of 105 mm and evaporation of 1315 mm [24, 33]. Soil salinity is high, alkalinity is strong, the average electrical conductivity of shallow soils (0–10 cm) is 5.41 mS/cm, with a pH value of 8.77. The average soil density is about 1.38 g/cm^3^ [24]. The natural plant populations were composed of drought-tolerant and salt-tolerant species, including *Populus euphratica*, *Haloxylon ammodendron*, *Tamarix ramosissima*, *Kalidium foliatum*, *Nitraria tangutorum*, *Alhagi sparsifolia*, *Apocynum venetum*, *Phragmites australis*, *Suaeda microphylla*, and *Salsola sinkiangensis*.

### Soil collection

**Fig 1:**
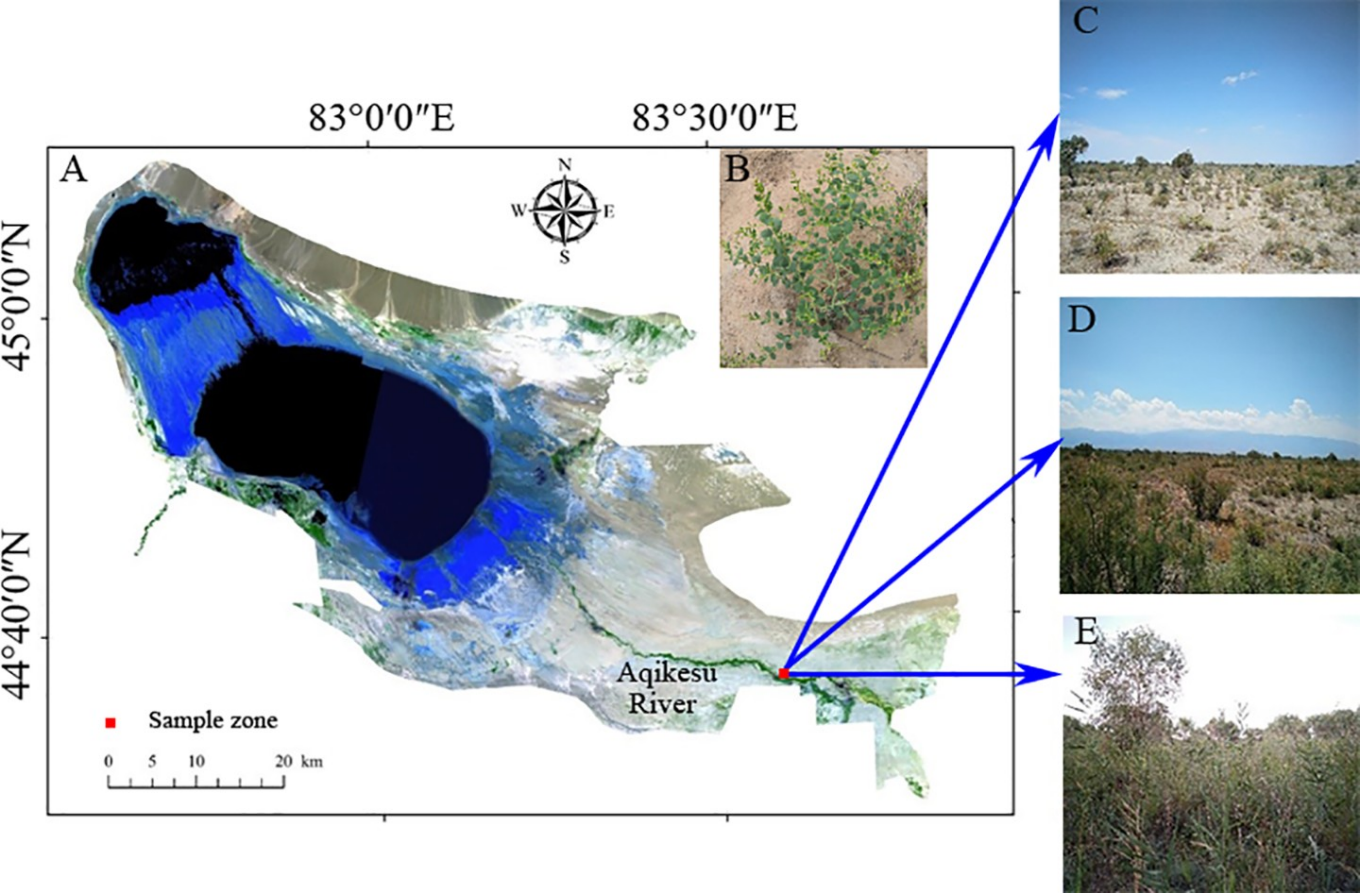
Schematic diagram of study area. (Autonomous Region is downloaded from The Gateway to Astronaut Photography of Earth website (https://eol.jsc.nasa.gov/SearchPhotos/). Because the map downloaded from this website is free and open to scholars, our study does not need to supply a copyright notice).

The sampling region was at the north side of Aqikesu River. The soil water content and total salt content gradually decreased with the increase of distance from the riverbank [34]. Three 10 m × 10 m plots were selected along the water and salt gradient from high to low: H (high water and salt), M (medium water and salt), L (low water and salt) (Fig 1). The three plots covered a distance of about 1.2 km. Soil samples were collected from Ebinur National Nature Reserve in August 2017. In each sampling plot, three healthy *Alhagi sparsifolia* individuals of a similar size were collected. The soil physicochemical properties followed the order: H plot (high water 8.39%–12.46% and salt 6.28%–8.10g/kg) > M plot (medium water 4.93%–7.08% and salt 3.14%–6.06g/kg) > L plot (low water 3.08%–5.34% and salt 2.36%–3.04g/kg). The average heights of the *Alhagi sparsifolia* individuals in the H plot, M plot, and L plot were 69 cm, 43 cm, and 32 cm, respectively. Soils were collected on a sunny day without precipitation during the preceding three days. We dug out the roots of the plant, shook off the loosely attached soils (as rhizosphere soil), and transferred them into 50-ml sterile centrifuge tubes. Bulk soil samples were collected from sites 30–40 cm away from each plant individual; fresh soils (about 50 g) were collected to a depth of approximately 40 cm and stored in a sterile plastic bag. These soils were immediately transported back to the lab on ice for DNA extraction. All soils were subdivided into two parts, one used for microbial community analysis, the other one for physical and chemical properties determination after natural drying, grinding, and sifting. In each plot, three rhizosphere and three bulk soils were collected for a total of 18 samples that were used in this study.

### Soil chemical analysis

Soil water content (SWC) was determined by a drying method: soil samples were dried in a hot air oven at 105°C for 48 hours. Soil pH was determined using the pH meter by the microelectrode method [35]. Conductivity (EC) was determined by the conductivity method [36]. Soil organic carbon (SOC) was determined by a potassium dichromate bulk density method. Total nitrogen (TN) was determined by a K_2_Cr_2_O_4_–H_2_SO_4_ digestion method. NH_4_^+^ was determined by indophenol blue colorimetry, while NO_3_^-^ was determined by phenoldisulfonic acid colorimetry. Available nitrogen (AN) was determined by the alkaline hydrolysis diffusion method. Total phosphorus (TP) was determined by the HClO_4_–H_2_SO_4_ method. AP was determined colorimetrically by ammonium molybdate. SO_4_^2-^ was determined by EDTA indirect complexometric titration, and Mg^2+^ was determined by atomic absorption spectrometry [37].

### DNA extraction, amplification, and sequencing

Total genome soil DNA was extracted using the CTAB/SDS method. DNA concentration and purity was monitored on 1% agarose gels. According to the concentration, DNA was diluted to 1 ng/μl using sterile water. The variable region (V4) of 16S rRNA genes was targeted using PCR primers 515F and 806R with a barcode on the forward primer. The fungal ITS1 region was amplified with primers ITS5–1737F and ITS2–2043R. PCR products were detected using electrophoresis on 2% agarose gel. Samples with a bright main strip between 400–450 bp were chosen for further experiments. PCR products were mixed in equimolar concentrations. Then, the mixture PCR of products was purified using a GeneJET Gel Extraction Kit (Thermo Fisher Scientific). Sequencing libraries were generated using NEB Next® Ultra™ DNA Library Prep Kit for Illumina (NEB, USA) following manufacturer’s recommendations and index barcodes were added. The library quality was assessed on the Qubit@ 2.0 Fluorometer (Thermo Fisher Scientific) and Agilent Bioanalyzer 2100 system. The library was sequenced on an Illumina HiSeq platform and 250-bp paired-end reads were generated. All the raw data generated in this study were deposited into the NCBI Sequence Read Achieve database under the accession number PRJNA707263 and PRJNA707265.

### Bioinformatics analysis

Illumina MiSeq sequencing generates raw double-ended sequence data and stores it in FASTQ format. Ambiguous, homologous and long sequences were removed using TrimMomatic software to remove the original two-ended sequences, and then the two ended sequences were merged using Flash software [38]. At the same time, UCHIME was used to detect and remove chimeric sequences [39]. The filtered sequences were then clustered into an operational taxonomic unit (OTU) at an identity threshold of 97%. A representative sequence from each OTU was selected for both taxonomic annotation using the Ribosomal Database Project (RDP) classifier [40] and BLAST against the NCBI and Silva databases [41]. OTUs with an RDP classification threshold below 0.8 or with identity and coverage lower than 90% were marked as unclassified. Singletons and sequences aligning to the mitochondria or chloroplast were removed. Rarefaction curves, richness, ACE, and Shannon index were used to evaluate alpha diversity using QIIME software (Version 1.7.0) [42].

### Data analysis

The mean and standard deviations of the soil physicochemical properties and the alpha diversity index of different samples were calculated. The Shapiro-Wilk and Levene’s tests were used to test the normality and homoscedasticity of data, respectively. The soil physicochemical properties and the alpha diversity index were log-transformed to satisfy the normal distribution. An analysis of variance (ANOVA) (Fisher’s least significant difference (LSD) test, *P* < 0.05) was used to determine whether there were significant differences between soil physicochemical properties, OTU richness, and alpha diversity index in the three plots. A paired t test (*P* < 0.05) was used to estimate significant differences between the rhizosphere and bulk soils in the same plots. The relative abundance were the ratios of the absolute abundances of the species to the total microbial abundance. The result was accepted as significant for *P* < 0.05. We used Spearman’s rank correlations to analyze the relationships between bacterial and fungal community compositions and soil environmental factors. We employed QIIME to calculate Unifrac distances and construct UPGMA cluster trees of soil samples. The structures of the bacterial and fungal communities were visualized by principal coordinates analyses (PCoA) based on weighted Unifrac distance. The variance partitioning analysis (VPA) focuses on the explaining amount of each environmental factor on the distribution of microbial community, so as to get the contribution degree of each environmental factor to the difference in microbial community distribution; then, the relative importance sorting method was used to normalize the contribution of soil environmental factors to the composition of soil bacterial and fungal communities.

The above statistical analyses were conducted and presented using the statistical package SPSS (PASW statistics 19.0; IBM Corporation, Armonk, NY, USA) and graphical software Origin (ver. 8.0; OriginLab, Northampton, MA, USA). Correlation analysis, PCoA and VPA were all done using the software R (ver. 3.6.1; R Development Core Team; www.r-project.org/).

## Results

### Soil physiochemical properties

The environmental factors of rhizosphere and bulk soil samples of *Alhagi sparsifolia* in the three H (high water and salt), M (medium water and salt), and L (low water and salt) plots in the study area are shown in Table 1. The soil pH is between 7.59 and 8.30 and is alkaline. The variance analysis method was used to analyze the environmental factors, and there was significant difference in SOC, TN and AN between rhizosphere soil and bulk soils (*P* < 0.05). However, in the rhizosphere soil of the three plots, there were significant differences between the SAL, SOC, TN, TP, and AP (*P* < 0.05); environmental factors SWC, NO_3_^-^, AN were significantly different between the H plot and the M and L plots (*P* < 0.05), but there was no significant difference in the pH, NH_4_^+^, and Mg^2+^ among the three gradients (*P* > 0.05). For bulk soils, there were significant differences in the soil environmental factors SAL, pH, and TP in the three plots (*P* < 0.05), and environmental factors SWC, AN, AP were significantly different between the H plot and the M and L plots (*P* < 0.05); NH_4_^+^, NO_3_^-^, SO_4_^2-^, and Mg^2+^ were not significantly different between the three plots (*P* > 0.05). In general, the physical and chemical properties of the soil in the H plot were characterized by the high water-salt content and high soil nutrient content, while the low water-salt content and lack of nutrients were common in the L plot.

**Table 1:** The physiochemical properties of rhizosphere and bulk soils associated with *Alhagi sparsifolia*.

Name	SWC (%)	SAL (g/kg)	pH	SOC (g/Kg)	TN (mg/Kg)	NH_4_^+^ (mg/Kg)	NO_3_^-^ (mg/kg)	AN (mg/Kg)	TP (mg/Kg)	AP (mg/kg)	SO_4_^2-^ (g/Kg)	Mg^2+^(g/Kg)
H.AS.R	9.91 ± 1.29^A^	7.84 ± 0.20^A^	8.13 ± 0.10^A^	5.43 ± 0.25^A^	390.16 ± 40.50^A^	0.88 ± 0.25^A^	11.13 ± 3.50^A^	25.08 ± 3.25^A^	617.51 ± 13.67^A^	29.21 ± 1.76^A^	14.30 ± 2.30^A^	0.15 ± 0.01^A^
H.AS.N	10.22 ± 1.06^a^	6.70 ± 0.22^a^	8.30 ± 0.07^a^	3.72 ± 0.84^a^	252.96 ± 40.81^a^	1.00 ± 0.37^a^	3.75 ± 1.65^a^	14.00 ± 1.31^a^	579.80 ± 26.40^a^	23.95 ± 2.51^a^	15.30 ± 3.86^a^	0.15 ± 0.05^a^
M.AS.R	6.17 ± 0.64^B^	4.51 ± 0.82^B^	7.73 ± 0.36^A^	4.06 ± 0.48^B^	243.46 ± 23.26^B^	1.18 ± 0.23^A^	0.74 ± 0.10^B^	14.58 ± 0.34^B^	382.95 ± 6.15^B^	18.98 ± 1.21^B^	9.31 ± 1.51^AB^	0.18 ± 0.06^A^
M.AS.N	5.60 ± 0.45^b^	3.61 ± 0.33^b^	7.95 ± 0.10^b^	2.96 ± 0.32^ab^	197.90 ± 21.54^ab^	1.02 ± 0.25^a^	0.83 ± 0.27^a^	6.42 ± 0.32^b^	372.28 ± 10.93^b^	17.17 ± 1.08^b^	9.03 ± 0.45^a^	0.11 ± 0.01^a^
L.AS.R	4.63 ± 0.78^B^	2.78 ± 0.10^C^	7.59 ± 0.13^A^	1.58 ± 0.22^C^	142.33 ± 13.77^C^	0.93 ± 0.28^A^	1.22 ± 0.45^B^	10.58 ± 0.64^B^	277.18 ± 17.90^C^	14.05 ± 0.33^C^	6.88 ± 0.62^B^	0.08 ± 0.01^A^
L.AS.N	3.83 ± 0.20^b^	2.65 ± 0.20^c^	7.67 ± 0.04^c^	1.37 ± 0.14^b^	127.24 ± 12.91^b^	0.94 ± 0.16^a^	1.17 ± 0.16^a^	3.50 ± 0.32^b^	300.47 ± 7.61^c^	15.14 ± 0.30^b^	9.01 ± 0.24^a^	0.12 ± 0.03^a^

Note: H represents (high water and salt); M represents (medium water and salt); L represents (low water and salt), AS represents *Alhagi sparsifolia*; R, rhizosphere soil; and N, bulk soil. Environmental factors: SWC, soil water content; SAL, soil salinity; SOC, soil organic carbon; TN, total nitrogen; NH_4_^+^, ammonium nitrogen, NO_3_^-^, nitrate nitrogen; AN, available nitrogen; TP, total phosphorus; AP, available phosphorus; SO_4_^2-^, sulfate ion; Mg_2_^+^, magnesium ion

### Diversity and structure of microbial communities

**Fig 2:**
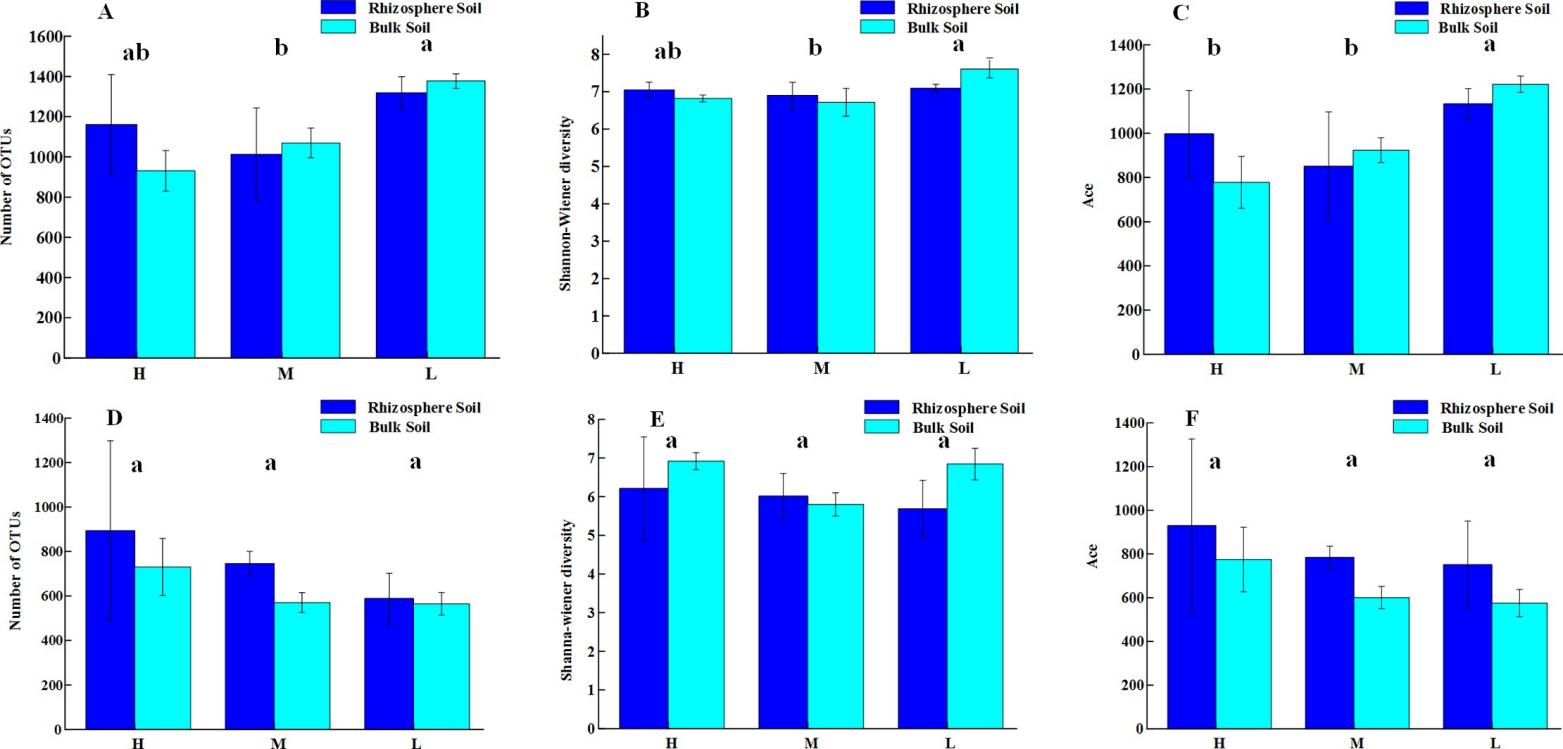
Alpha diversity of bacterial and fungal communities in rhizosphere and bulk soils associated with *Alhagi sparsifolia*.

A total of 1,293,969 high quality bacterial sequences were obtained from all soil samples, from which 20,608 OTUs were identified. Among the three plots, there was a significant difference in the bacterial OTU sequences between the M and L plots (*P* < 0.05), with the largest number of OTUs in the L plot (Fig 2). The Shannon-Wiener index significantly differed between the M and L plots (*P* < 0.05), and the diversity was highest in the L plot. The OTU richness in the L plot was significantly higher than that in the H and M plots. A total of 1,448,470 high-quality fungal sequences were obtained, from which 12,281 OTUs were identified. There was no significant difference in fungal community diversity among the three plots.

**Fig 3:**
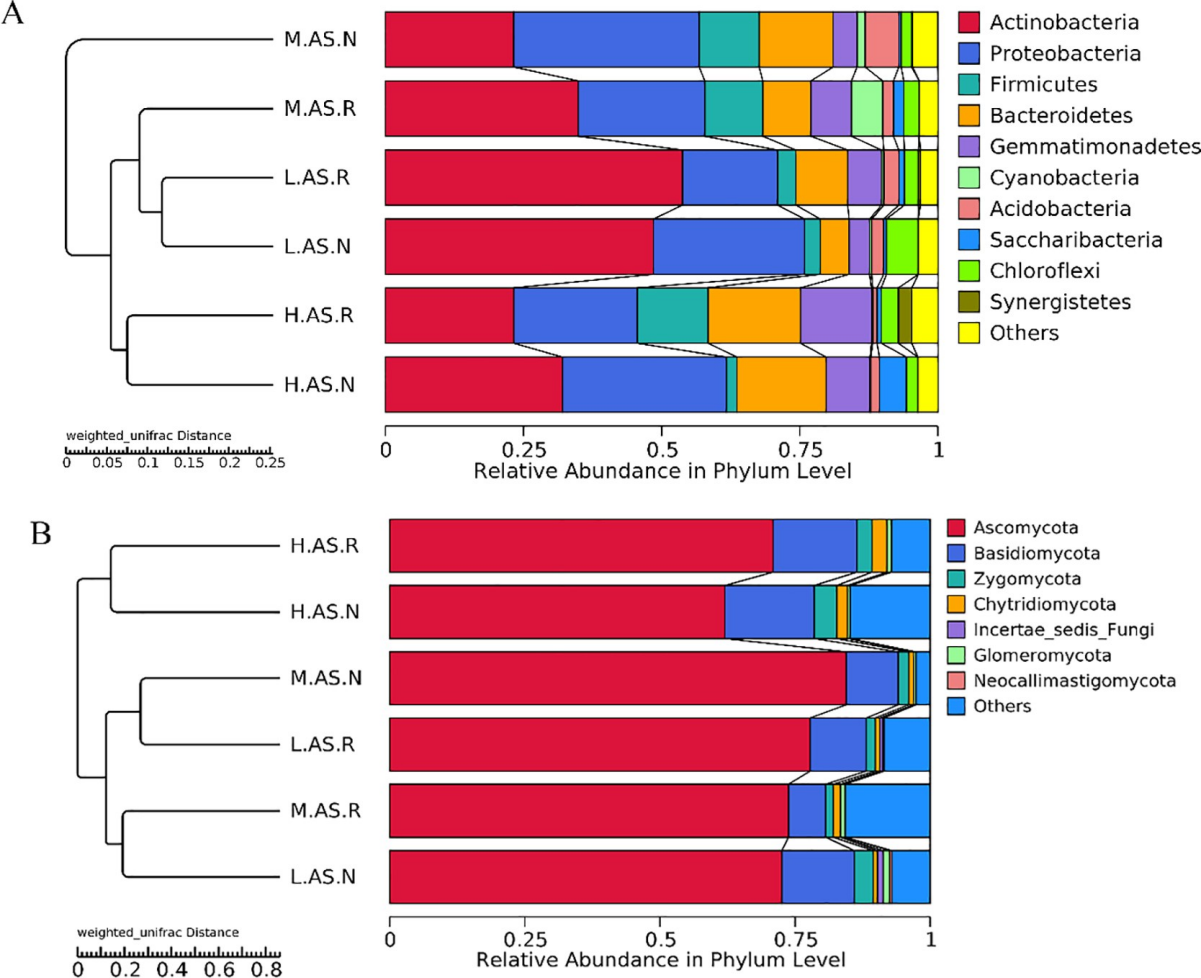
Diagrams depicting community composition and relative abundance at phylum levels: (A), bacteria; (B), fungi.

All of the bacterial OTUs belonged to 643 genera, 267 families, 104 classes, and 46 phyla. The abundant phyla were Actinobacteria, Proteobacteria, Firmicutes, Bacteroidetes, Gemmatimonadetes, Cyanobacteria, and Acidobacteria (Fig 3). The relative abundances of the dominant bacterial phylum Actinobacteria in the rhizosphere and bulk soils in the H plot were 23.3% and 32.0%, in the M plot were 34.9% and 23.2%, and in the L plot were 53.8% and 48.5%, respectively. The relative abundances of Proteobacteria ranged from 17.2% to 33.6% among the three plots; the relative abundance of Proteobacteria in the bulk soil was higher than in the rhizosphere soil, and the relative abundances of Proteobacteria were the highest in M plots, followed by the H and L plots. The relative abundances of Firmicutes were 12.8% and 1.9%, respectively, in the rhizosphere and the bulk soils of the H plot. Bacteroidetes abundances in the rhizosphere and bulk soils in the H plot were 16.7% and 16.1%, in the M plot were 8.7% and 13.4%, and in the L plot were 9.3% and 5.2%, respectively. It can be seen from the cluster diagram that the composition of the bacterial community structure in rhizosphere and bulk soil is similar at the phylum level, and the community structure of bacteria in the H plot soil shows its uniqueness.

Seven phyla, 30 classes, 253 families, and 455 genera were identified in the fungal phylum from all the soil samples. Ascomycota was the dominant phylum, but its relative abundance varied in the three gradients, as well as by soil types. The relative abundance of Ascomycota was higher in the M plot than in the other two plots, where its abundance was 73.8% in rhizosphere soil and 84.5% in bulk soil. In H plot soils, Ascomycota accounted for 71.0% and 62.0% of all OTUs in rhizosphere and bulk soil, respectively, while it was 7.8% and 72.5% in the L plot. The relative abundance of Basidiomycota was highest in the H plot, with 15.5% in the rhizosphere soil and 16.6% in the bulk soil, while lowest in the M plot with relative abundance of 6.9% and 9.6%, respectively. Zygomycota showed a similar pattern with Basidiomycota.

### Correlations between community composition and soil properties

**Fig 4:**
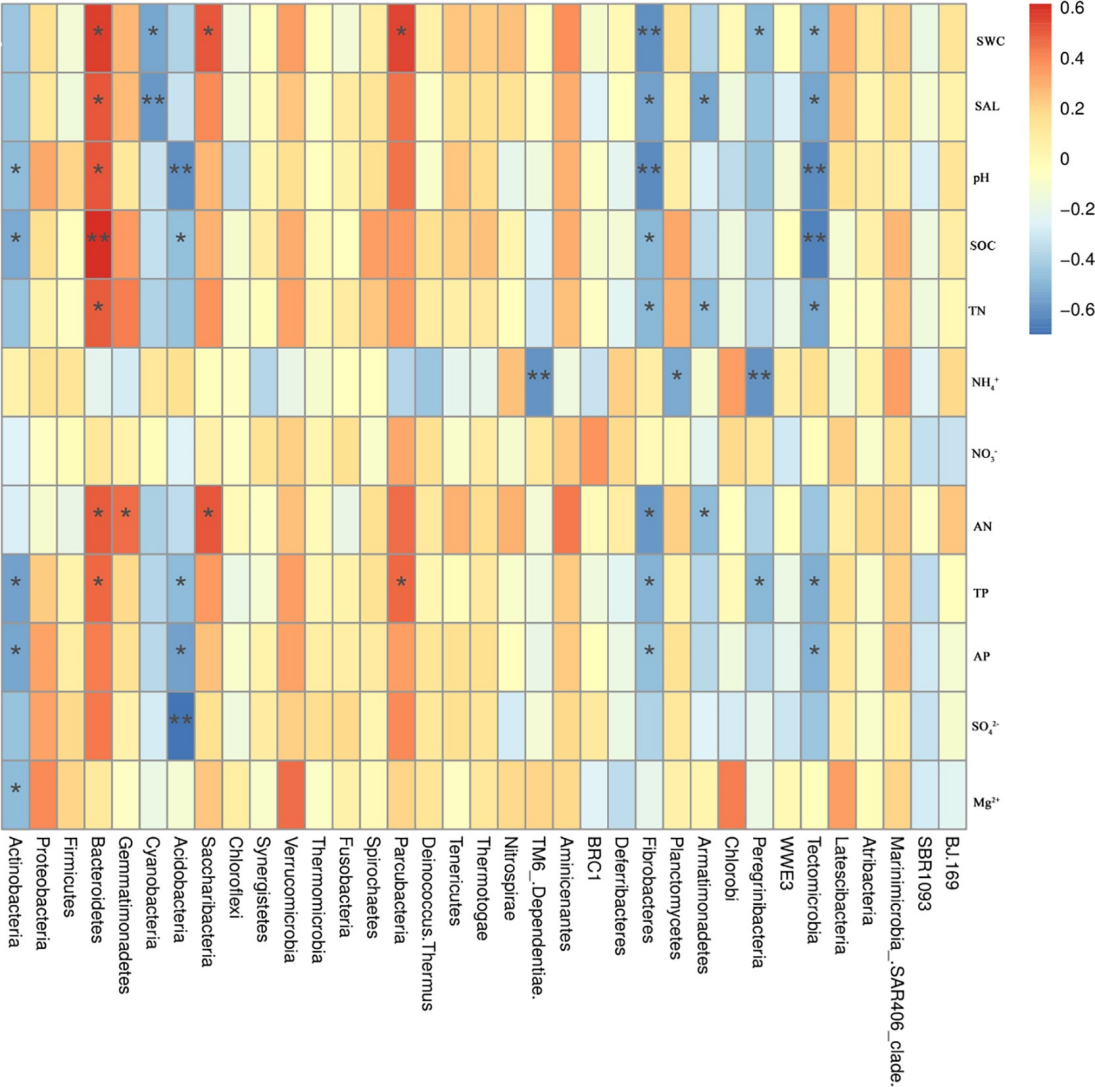
Correlation analysis between bacterial community composition and environmental factors.

The Spearman rank correlation was used to analyze the relationship between environmental factors and the abundance of dominant bacteria and fungi populations. The correlation heat map showed that Actinobacteria was significantly negatively correlated with soil pH, SOC, TP, AP, and Mg^2+^ (*P* < 0.05), with correlation coefficients of -0.49, -0.54, -0.56, -0.55, and -0.48, respectively. However, Actinobacteria was positively correlated with ammonium nitrogen. Firmicutes had a negative correlation with water and salt. Bacteroidetes showed a significant positive correlation with soil SOC (*P* < 0.01) with a correlation coefficient of 0.61. Bacteroidetes showed a significant positive correlation with SWC, SAL, pH, TN, AN, and TP (*P* < 0.05), with correlation coefficients of 0.57, 0.52, 0.50, 0.51, and 0.48, respectively. However, Bacteroidetes was negatively correlated with ammonium nitrogen. Gemmatimonadetes was positively correlated with AN (*P* < 0.05, correlation coefficient = 0.47). Cyanobacteria had a significant negative correlation with SAL (*P* < 0.01) and SWC (*P* < 0.05), with correlation coefficients of -0.59 and -0.55, respectively. There was no significant correlation between the composition of most bacterial groups and soil physical and chemical factors (Fig 4).

**Fig 5:**
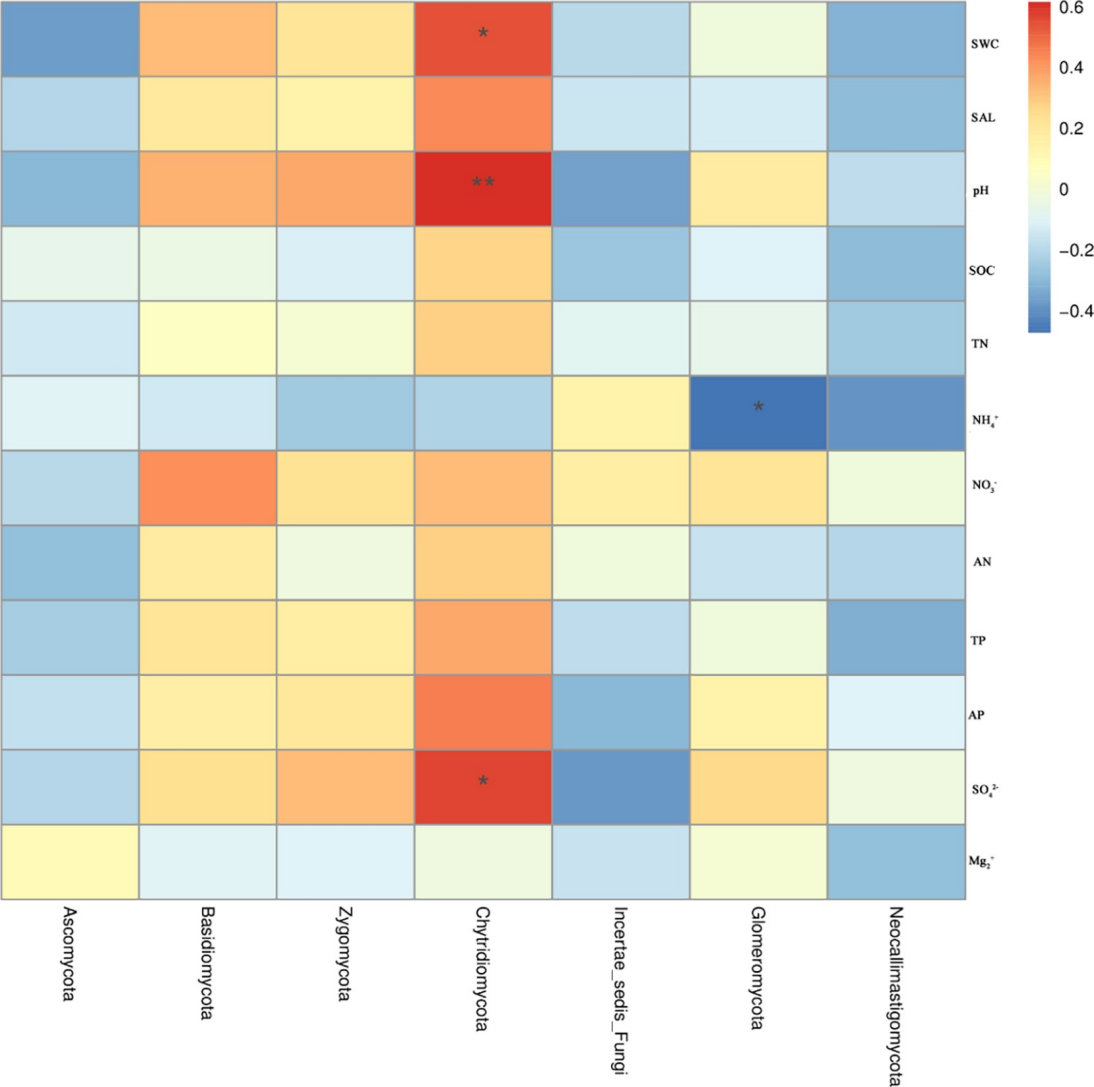
Correlation analysis between fungal community composition and environmental factors.

The dominant fungal phylum Ascomycota was negatively correlated with physical and chemical parameters, while Basidiomycota was positively correlated with SWC, SAL, pH, NO_3_^-^, AN, TP, AP, SO_4_^2-^, and negatively correlated with SOC and NH_4_^+^. Chytridiomycota was significantly positively correlated with pH (*P* < 0.01), significantly positively correlated with SWC, AP, SO_4_^2-^ (*P*< 0.05), and negatively correlated with NH_4_^+^ (Fig 5).

### Relationship between microbial community structure and soil physiochemical parameters and their contributions

**Fig 6:**
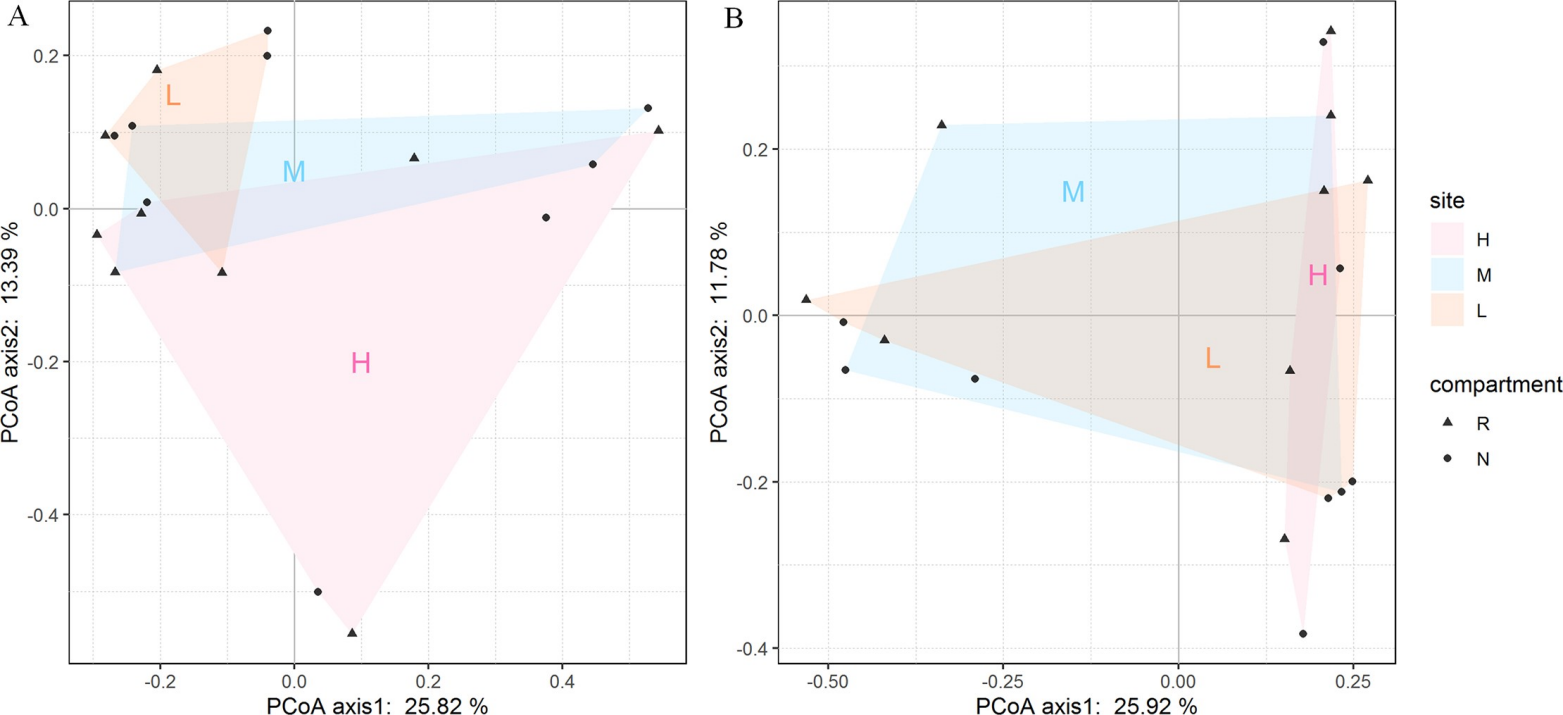
PCoA analysis of rhizobacteria communities was based on Bray-Curtis distance at OTU level. H (high water and salt), M (medium water and salt), L (low water and salt). R (rhizosphere soil), N (bulk soil).

The PCoA for bacterial (Fig 6A) and fungal (Fig 6B) communities, based on the weighted Unifrac distance of OTUs, did not display a clear separation of samples both water/salt gradients and soil compartments. Therefore, we continue to explore the role of major environmental factors in shaping soil microbial community structure.

**Fig 7:**
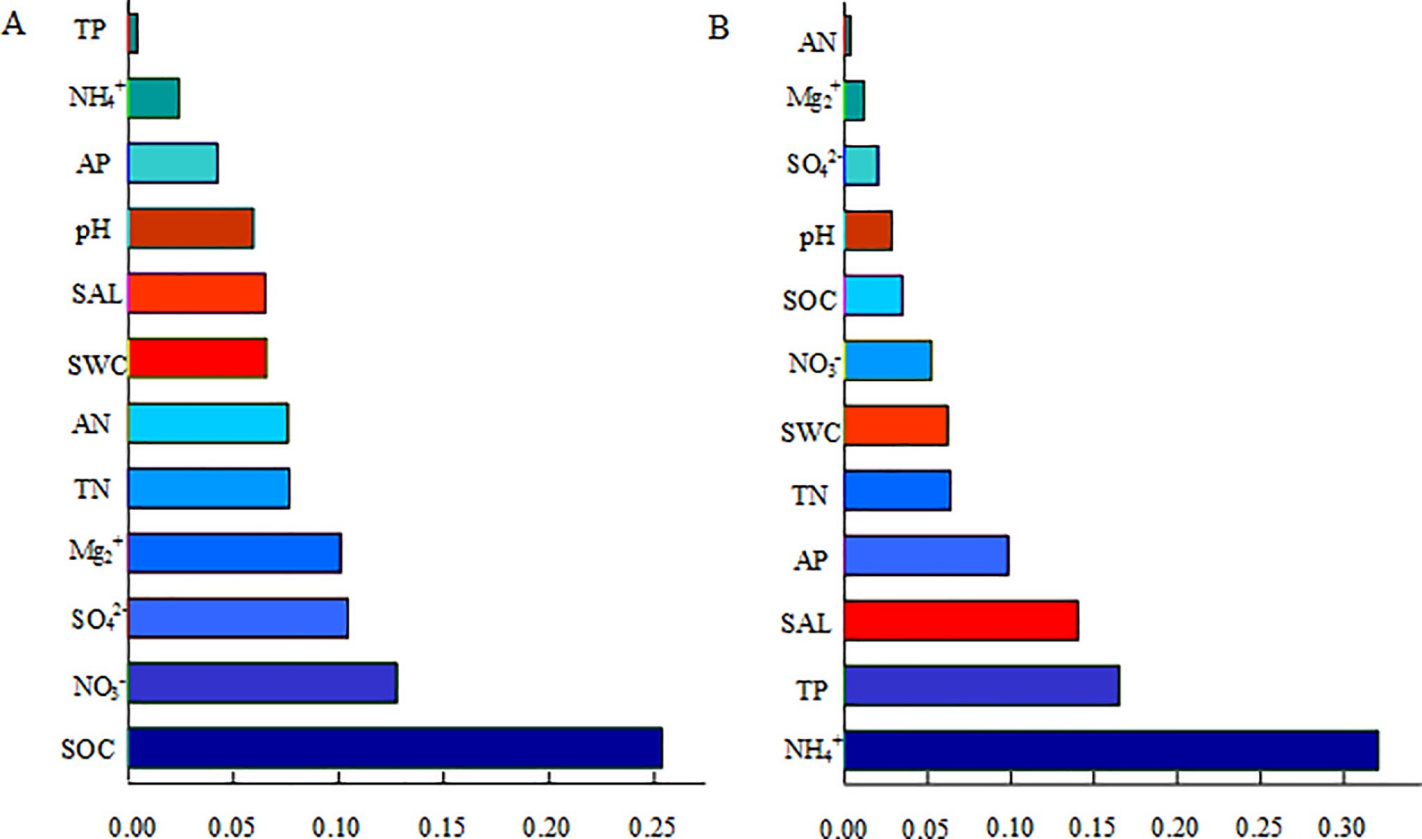
Sequence of the influence of environmental factors on soil microbial community structure of the bacterial community (A) and fungal community (B).

Results based on variation partitioning analysis found that SOC was the main environmental factor affecting the bacterial community structure (explaining 25.38% of the variation), followed by nitrate nitrogen and SO_4_^2-^ (explaining 12.74% and 10.44% of the variation, respectively). For the fungal community, the ammonium nitrogen explained 25.38% of the variation, followed by TP (16.49%) and SAL (14.02%). These results indicated the main driving factor of bacterial community structure in Ebinur Lake Basin was organic matter SOC, while ammonium nitrogen was the primary environmental factor affecting the fungal community structure. (Fig 7).

**Fig 8:**
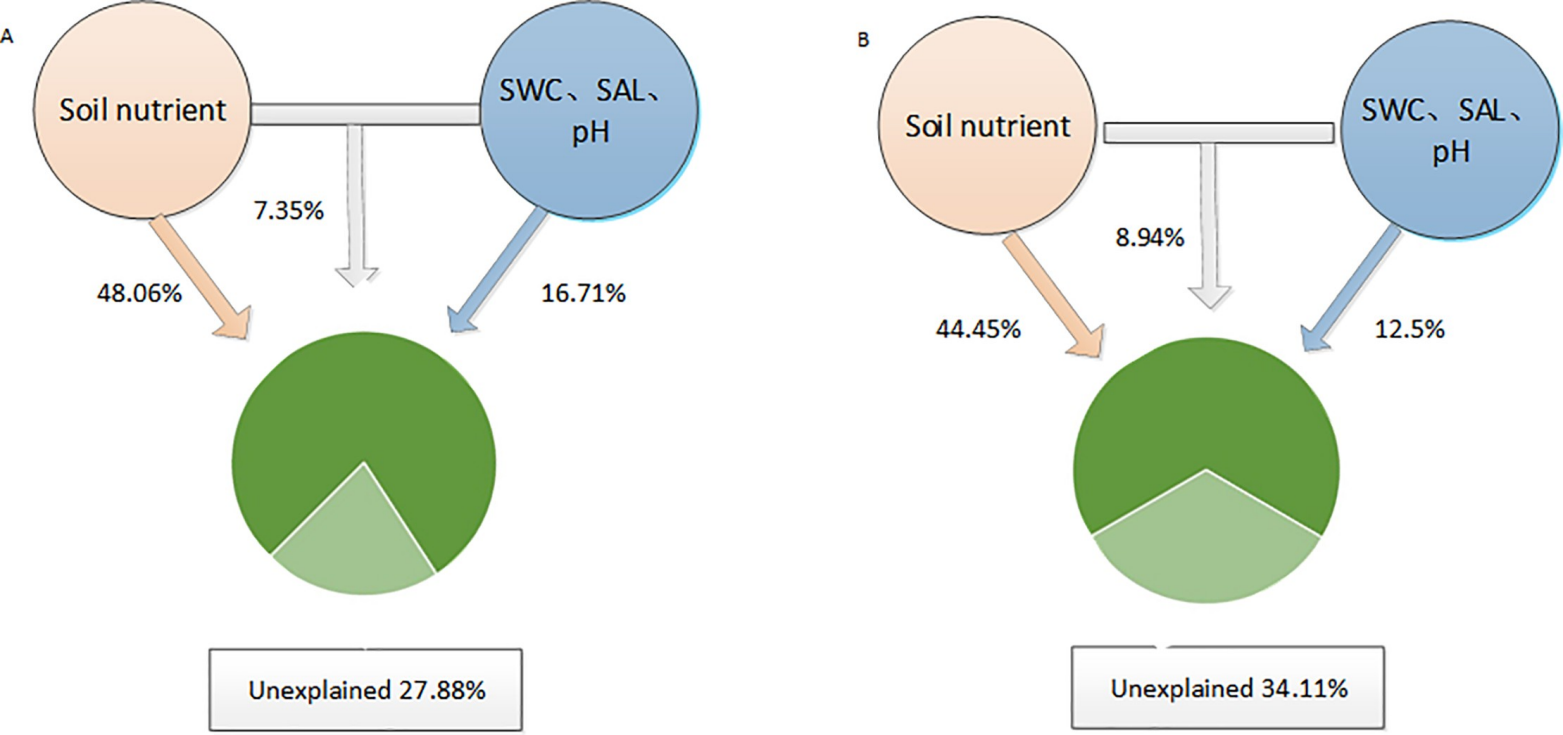
The variance partitioning analysis (VPA) for the soil microbial community with soil nutrients and soil water content (SWC), soil salinity (SAL) and pH: (A), bacterial community; (B), fungal community.

The VPA showed that soil nutrients, SWC, SAL, and pH explained 72.12% and 65.89% of the variation in the bacterial and fungal community structures, respectively. As a whole, soil nutrients explained 48.06% of the variation, with SWC, SAL, and pH together explaining 16.71% of the variation, and the mutual explanatory amount of the two further explaining 7.35%. The soil nutrients accounted for 44.45% of the variations in the fungal community, with SWC, SAL, and pH explaining 12.50% of the variance, and the mutual explanatory amount of the two accounted for 8.94% (Fig 8). By comparison, VPA showed that soil nutrients explained more than SWC, SAL, and pH of the differences in microbial community structure. These results suggest that soil nutrients might play a more important role in shaping microbial community structure and distribution than SWC, SAL, and pH.

## Discussion

### Dominant soil microbiomes associated with *Alhagi sparsifolia*

The soil microbial community is the main decomposer in terrestrial ecosystems, and very important to maintaining soil ecological function. We found that the dominant bacteria associated with the halophyte *Alhagi sparsifolia* were Actinobacteria and Proteobacteria in the rhizosphere and the bulk soil, which was consistent with previous research in desert ecosystems [43–45]. In general, the Acidobacteria had higher abundances in acidic soils [17]; in the desert area around Ebinur Lake Basin, the soil is alkaline (pH 7.59–8.30), and the average abundance of Acidobacteria in our three plots was more than 1%, which is lower than that of the other dominant phylum. Xiong et al. (2012) also found the existence of Acidobacteria in alkaline soil [46], which shows that Acidobacteria has a certain tolerance to higher soil pH. Acidobacteria also mostly exist in soil environments with poor nutrition [47]; soil nutrient deficiency in arid areas is further demonstrated by Sun et al. (2013) that some bacteria [48], such as Acidobacteria and Proteobacteria, could be used as indicators of soil nutrient status.

The number of fungal species in the desert is less than that of bacteria, but fungi play an indispensable role as they can release biologically-available nutrients; combined with desert plants, fungi also enhance their colonization and development [49]. We found that the total abundance of Basidiomycota and Ascomycota in our three plots was higher than 75%, with a relative abundance of Ascomycota of more than 60%. Most fungi in desert soil are closely related to vegetation. Most members in Ascomycota are saprophytes, which play an important role in degradation of soil organic matter, which is consistent with previous research on Ascomycota in dryland soil communities [50].

### Factors influencing soil microbial diversity and community structure

Soil environmental factors not only affect the bacterial community diversity, but also the distribution characteristics of the bacterial community. Soil water content (SWC) is an important limiting factor of soil microbial activity in arid and semi-arid environments. This study found that Actinobacteria was the main phylum, significantly higher in L (low water and salt) plots than in the H (high water and salt) and M (medium water and salt) plots, which may be due to their ability for sporulation to reduce the damage caused by drought, high temperature, and radiation, and a competitive advantage through the synthesis of secondary metabolites, so they can survive in desert areas with sparse vegetation for a long time [51]. We also saw a significant positive correlation between Chytridiomycota and SWC, which was consistent with the results of Raúl et al. (2018) [52]. Taniguchi et al. (2012) found that when the soil moisture ranged from 0% to 15%, the diversity of soil bacterial and fungal communities in arid and semi-arid areas showed a significant positive correlation with the soil moisture [53].

Soil salinity is an important limiting factor for the soil microbial community, which has a significant impact on the structure and diversity of soil bacterial community [54]. Among the three water/salt gradients, in the L plot, the bacterial OTU richness and Shannon Wiener Index reached the maximum. The reason for the highest diversity under low water and low salt may be that salt has a significant inhibitory effect on soil bacterial microorganisms, and the inhibitory effect of salt is significantly higher than the promotion of water and nutrients on bacterial microorganisms. This is consistent with other studies in desert ecosystems (Zhang et al. 2019) showing that salt is the key factor shaping the diversity of desert ecosystems [27]. The nutrient content of salinized soil in arid areas is low, and soluble salt rise from the bottom layer along the soil capillary system to the surface soil, resulting in salinization of the surface soil [55], which is not conducive to the growth and reproduction of soil microorganisms. Xiong et al. (2015) found that Firmicutes was positively correlated with SAL, which is consistent with the results of this study [56]. Therefore, the results of this study further indicated that soil SAL may be an important factor leading to changes in soil bacterial communities. With the increase of SAL in soil, the abundance of Bacteroides increased, for halophytes, which may be due to the increase of SAL to promote an increase in the number of salt tolerant or halophilic bacteria. However, the influence of salinity on fungi is not significant, which may be because the fungal chitin cell wall has a good protective effect on water loss caused by low water content, and can therefore improve the fungal resistance to low water potential caused by high salinity [28].

Many studies have shown that pH is a key factor affecting the distribution of microbial community structure [6, 57]. One previous study revealed a significant correlation between the relative abundance of Bacteroides and Acidobacteria and pH, but no significant correlation between Proteobacteria and pH [6]. This study also found that Bacteroides and Acidobacteria had a significant positive correlation and an extremely significant negative correlation with pH, respectively. In addition, there was a significant positive correlation between Actinobacteria and pH in other research, but Actinobacteria were not sensitive to pH in this study. Although pH has a significant impact on the overall microbial community in this study area, the relative abundances of the microbial community at the phylum levels had no significant correlations with pH. These results indicate that pH may not be a main factor affecting the relative abundance of these microorganisms, as soil salinity and nutrient elements such as carbon or nitrogen have more restrictive effects on these microorganisms. In contrast, soil pH does not directly affect the community structure of microorganisms, but does have an effect as a comprehensive variable index. Other physical and chemical properties of the soil, such as the utilization of available nitrogen, phosphorus, soil organic carbon, and metal ions, are directly or indirectly related to pH [6].

### The main driving factors of soil microbial community structure

Based on the contribution of each factor analysis and sequencing, SOC was the main environmental factor affecting the bacterial community structure of *Alhagi sparsifolia* soils, accounting for 25.38% of the total variation in community structure. This result shows that the organic carbon content has the most direct impact on the bacterial community structure, which is consistent with Maestre et al (2015) [50]. Compared with the fungal community, SOC has more influence on the bacterial community. As an extremely important link between soil and plants, microorganisms promote the transformation and circulation of soil organic matter and soil nutrients, and are one of the important indicators for evaluating soil fertility [58]. NO_3_^-^ is the secondary environmental factor that affects the bacterial community structure, while NH_4_^+^ is the most important environmental factor affecting the fungal community structure, accounting for 32.06% of the total interpretation. It can be concluded that the soil nitrogen is one of the most important factors affecting the soil microorganisms of *Alhagi sparsifolia*. As a nitrogen-fixing plant, *Alhagi sparsifolia* can produce stronger stimulation effects and improve the rhizosphere with nodulation and nitrogen fixation through the role of nitrogen fixation by rhizobia; the content of soil organic matter can improve the soil nutrient status and promote microbial activity [59, 60].

Bacterial and fungal communities are driven by different forms of nitrogen. Bacterial structure is mainly affected by nitrate nitrogen, while fungal structure is more influenced by ammonium nitrogen. Microorganisms can also absorb nitrate nitrogen from the soil, but since the nitrogen in nitrate nitrogen is oxidized, it needs to be reduced to ammonium nitrogen after entering the cell to further participate in the synthesis of organic molecules. Therefore, the use of nitrate nitrogen by microorganisms requires more energy than ammonium nitrogen, and the microorganisms prefer the absorption of ammonium nitrogen. The growth characteristics of soil fungal hyphae and the efficient use of nitrogen (nitrogen can be transferred between different parts of the hypha) give the fungi the ability to actively seek nitrogen resources [61]. Therefore, the microbial groups in arid and low nutrient ecosystems have potential nutritional preference, which adapts to environmental stresses. This is also supported from the viewpoint of gene level transfer and niche differences [62]. TP is the second largest environmental factor affecting the fungal community, and nearly 90% of plant species can form arbuscular mycorrhizal associations in nature [63]. Arbuscular mycorrhiza can improve the symbiotic systems of higher plants and effectively promote the absorption of phosphorus by plants [59].

In the bacterial and fungal microbial communities, the VPA results show that the environmental variables explained a high degree of variation, and the soil nutrient contribution of bacteria and fungi was more than 40%. These results show that soil nutrients play a more important role in shaping the microbial community of desert soil than we previously thought, which can better explain the distribution pattern of the microbial community; this is consistent with previous research [64]. In the desert area of Ebinur Lake Basin, soil microorganisms have been adapted to the low rainfall and high salt alkali content of the local arid environment through long-term evolution. This research shows that SWC, SAL, and pH also have a strong influence on the bacterial microbial community. However, the sensitivity of fungal community structures to changes in the SWC, SAL, and pH was lower than that of the bacterial community. The superposition and interaction of SWC, SAL, pH, and soil nutrients were higher than that of the bacterial community, forming a unique distribution pattern in the fungal microbial community. The distribution of soil microorganisms under semi-arid ecosystem conditions in arid areas is not only affected by soil nutrients, but also by SWC, SAL, and pH. It has been found that soil water shortages can also restrict the transport and availability of soil nutrients, thus intensifying the relationship between plants and microorganisms in the nutrient-competitive microenvironment [65]. The lack of soil available nutrients and nutrient competition between the microbial communities can also lead to the degradation of ecosystem desert zones. Therefore, the growth and survival of microorganisms in soils are affected by various environmental factors, which determine the utilization of different resources by microorganisms.

## Conclusions

In the desert area of Ebinur Lake Basin, the diversity of bacteria in soil samples of *Alhagi sparsifolia* is relatively high. The most abundant bacterial phyla were Actinobacteria, Proteobacteria, Firmicutes, and Gemmatimonadetes. Under low water-salt gradients, the relative abundance of Actinobacteria was significantly higher than in the soils of the high and medium water-salt gradients. Ascomycota was the dominant fungal phylum, while Basidiomycete and Zygomycete were also abundant in the soil community. Among the three water/salt gradients, in the M plot (medium water and salt), the bacterial OTU richness, and Shannon Wiener Index reached the minimum. Soil microbial community structure was affected by soil nutrients and multiple other environmental factors. The driving forces and relative contributions varied between bacteria and fungi. Soil nutrients were the main contributors to the differences in the bacterial and fungal communities, explaining 48.06% and 44.45% of the variation, respectively. SOC and nitrate nitrogen were the main factors affecting the microbial community of soil bacteria, while the main driving forces of fungal community were ammonium nitrogen and TP. Bacterial and fungal community structure were differentially driven by the form of available nitrogen in the soil; bacterial community structure was mainly affected by nitrate nitrogen, while fungal community structure was mainly driven by ammonium nitrogen.

## Supporting information

S1 File (ZIP)
